# Enhanced chondrogenic differentiation of iPS cell-derived mesenchymal stem/stromal cells via neural crest cell induction for hyaline cartilage repair

**DOI:** 10.3389/fcell.2023.1140717

**Published:** 2023-05-10

**Authors:** Denise Zujur, Ziadoon Al-Akashi, Anna Nakamura, Chengzhu Zhao, Kazuma Takahashi, Shizuka Aritomi, William Theoputra, Daisuke Kamiya, Koichi Nakayama, Makoto Ikeya

**Affiliations:** ^1^ Center for iPS Cell Research and Application (CiRA), Kyoto University, Kyoto, Japan; ^2^ Center for Regenerative Medicine Research, Faculty of Medicine, Saga University, Saga, Japan; ^3^ Laboratory of Skeletal Development and Regeneration, Institute of Life Sciences, Chongqing Medical University, Chongqing, China; ^4^ Research Institute for Bioscience Product and Fine Chemicals, Ajinomoto Co., Inc, Kawasaki, Japan; ^5^ Takeda-CiRA Joint Program (T-CiRA), Kanagawa, Japan

**Keywords:** mesenchymal stem/stromal cells, induced mesenchymal stem/stromal cells, induced pluripotent stem cells, chondrogenesis, cartilage, chondrocytes, tissue engineering, cell-based therapy

## Abstract

**Background:** To date, there is no effective long-lasting treatment for cartilage tissue repair. Primary chondrocytes and mesenchymal stem/stromal cells are the most commonly used cell sources in regenerative medicine. However, both cell types have limitations, such as dedifferentiation, donor morbidity, and limited expansion. Here, we report a stepwise differentiation method to generate matrix-rich cartilage spheroids from induced pluripotent stem cell-derived mesenchymal stem/stromal cells (iMSCs) via the induction of neural crest cells under xeno-free conditions.

**Methods:** The genes and signaling pathways regulating the chondrogenic susceptibility of iMSCs generated under different conditions were studied. Enhanced chondrogenic differentiation was achieved using a combination of growth factors and small-molecule inducers.

**Results:** We demonstrated that the use of a thienoindazole derivative, TD-198946, synergistically improves chondrogenesis in iMSCs. The proposed strategy produced controlled-size spheroids and increased cartilage extracellular matrix production with no signs of dedifferentiation, fibrotic cartilage formation, or hypertrophy *in vivo*.

**Conclusion:** These findings provide a novel cell source for stem cell-based cartilage repair. Furthermore, since chondrogenic spheroids have the potential to fuse within a few days, they can be used as building blocks for biofabrication of larger cartilage tissues using technologies such as the Kenzan Bioprinting method.

## 1 Introduction

Cartilage tissue has limited self-healing capacity owing to the lack of blood vessels and insufficient blood supply necessary to promote cell proliferation and differentiation *in situ* (Ulrich-Vinther et al., 2003; Sophia Fox et al., 2009). Therefore, damaged cartilage is difficult to repair, and sound long-term therapeutic effects have not yet been obtained. Cell-based therapies have been explored as alternative approaches for treating cartilage defects. Primary chondrocytes and mesenchymal stromal/stem cells (MSCs) isolated from adult tissues are the most common cell sources used in regenerative medicine (Mardones et al., 2015). However, both cell types have limitations such as dedifferentiation, donor morbidity, and limited expansion, resulting in heterogeneous and inconsistent cell products and poor clinical outcomes.

Induced pluripotent stem cells (iPSCs) are promising alternatives to generate a large number of chondrogenic precursors for cell therapy (Boreström et al., 2014). The advantages of using iPSC-derived mesenchymal stromal/stem-like cells (iMSCs) over adult tissue-derived MSCs include extensive cell expansion *ex vivo* and the elimination of invasive biopsies. In addition, iMSCs can be genetically modified to increase their differentiation potential, reduce their immunogenicity, and introduce patient-specific mutations for research purposes and disease modelling (Zhao and Ikeya, 2018).

Chondrogenesis is a sequential process that occurs mainly during the embryonic stages. Chondrocytes are derived from mesenchymal precursors originating from the paraxial mesoderm, lateral plate mesoderm, and neural crest. The embryonic nature of iPSCs provides the opportunity to recapitulate the developmental path of chondrocyte differentiation. Several protocols have been established to generate chondrocytes and cartilage-like tissues from iMSCs (Umeda et al., 2012; Koyama et al., 2013; Rodríguez Ruiz et al., 2021; Rodríguez Ruiz et al., 2021).

Our team as well as others have successfully established strategies to generate iPSC-derived neural crest-like cells (iNCC) as an intermediate source of iMSCs (Lee et al., 2010; Menendez et al., 2011; Fukuta et al., 2014; Umeda et al., 2015; Umeda et al., 2015; Kamiya et al., 2022; Kamiya et al., 2022). We have previously shown that the resulting iMSCs are highly expandable, cryogenically preservable, and can differentiate into osteoblasts, chondrocytes, and adipocytes (Fukuta et al., 2014; Zhao and Ikeya, 2018). We have extensively explored the use of iMSCs in drug discovery and disease modeling (Zhao and Ikeya, 2018; Nakajima and Ikeya, 2019). Our efforts to generate cells for regenerative medicine prompted us to translate our protocol into a xeno-free system for generating functional iMSCs (Kamiya et al., 2022). We have demonstrated the *in vivo* therapeutic effect of iMSCs on musculoskeletal tissues, including muscle, bone (Kamiya et al., 2022), and laryngeal cartilage (Yoshimatsu et al., 2021).

Here, we attempted to extend the applications of iMSCs to regenerative medicine and tissue engineering by generating high-quality cartilage spheroids *in vitro*. Because successful neocartilage formation may depend on both the susceptibility of the precursor cells to chondrogenesis and the proper manipulation of the signaling pathways involved in chondrocyte commitment, our approach included screening the chondrogenic potential of two types of iMSCs and optimizing the chondrocyte differentiation strategy. Particularly, we propose the use of TD-198946 (TD), a small molecule used to enhance chondrogenic differentiation of various human progenitor cells, including BM-MSCs (Yano et al., 2013b), synovium-derived stem cells (Chijimatsu et al., 2019; Kobayashi et al., 2020), and nucleus pulposus cells (Kushioka et al., 2020).

## 2 Results

### 2.1 iMSCs display different differentiation potential towards chondrogenesis

**FIGURE 1:**
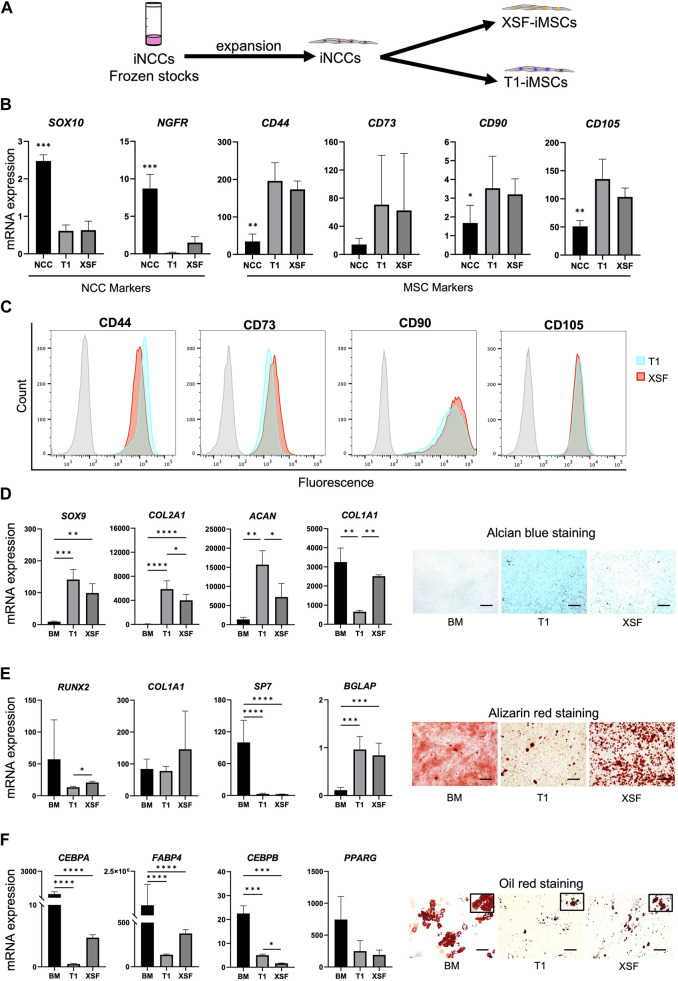
Characterization of iMSCs generated from expanded iNCCs with two culture media under xeno-free conditions **(A)** Schematic diagram of the induction protocol for iMSC differentiation from iNCCs. **(B)** mRNA expression of NCC and MSC markers determined by RT-qPCR in iMSCs generated using T1 and XSF media. Data is presented as folds relative to iPSCs, and are the means ± SD from four independent experiments. *p < 0.05, **p < 0.005, ***p < 0.001, ****p < 0.001 in NCC vs. all others. No significant difference was found in T1 vs. XSF for any marker. **(C)** Flow cytometry analysis of the distinctive MSC markers in the iMSCs generated using T1 and XSF media. **(D)** Characterization of BM-MSCs, T1-iMSCs and XSF-iMSCs differentiated to chondrogenic cells in micromass culture. mRNA expression levels of chondrocyte markers (left) and representative pictures of alcian blue staining (right) are shown, scale bar: 100 μm. **(E)** Characterization of BM-MSCs, T1-iMSCs and XSF-iMSCs differentiated to osteogenic cells at 3 weeks. mRNA expression levels of osteoblast markers (left) and representative pictures of alizarin red staining (right) are shown, scale bar: 100 μm. **(F)** Characterization of BM-MSCs, T1-iMSCs and XSF-iMSCs differentiated to adipogenic cells at 3 weeks. mRNA expression levels of adipocyte markers (left) and representative pictures of Oil Red O staining (right) are shown, scale bar: 100 μm. RT-qPCR data **(D)**, **(E)** and **(F)** is presented as folds relative to iPSCs, and are the means ± SD of three independent experiments (*n* = 3) for iMSCs and one experiment for BM-MSCs (*n* = 6). *p ≤ 0.05, **p ≤ 0.01, ***p ≤ 0.005, and ****p ≤ 0.0001 as indicated.

To establish an efficient differentiation strategy for generating cartilage-like tissue from iMSCs, we first studied the characteristics and differentiation potential of iMSCs generated from the iNCC lineage under different conditions. Cryopreserved iNCCs were differentiated and expanded using our previously established protocol (Kamiya et al., 2022) and were further differentiated into iMSCs using two types of xeno-free culture media, XSF and T1 media (Figure 1A). After three passages, iMSCs differentiated with T1 (T1-iMSCs) or XSF (XSF-iMSCs) were analyzed. The NCC markers *SOX10* and *NGFR* were downregulated, while the typical MSC markers (*CD44*, *CD73*, *CD90*, and *CD105*) were upregulated in both types of iMSCs compared to the iNCCs, and no significant differences were found between T1-iMSCs and XSF-iMSCs for any of the markers analyzed (Figure 1B). Flow cytometry analysis also confirmed that nearly all of the T1-and XSF-iMSCs expressed MSC marker proteins (CD44, CD73, CD90, and CD105) at comparable levels (Figure 1C). Next, we examined the three-lineage differentiation potential of the iMSCs. Human bone marrow-derived MSCs (BM-MSCs) were also differentiated as a reference. T1-and XSF-iMSCs were able to differentiate into chondrocytes, osteoblasts, and adipocytes (Figures 1D–F). However, T1-iMSCs showed superior chondrogenic potential compared to XSF-iMSCs and BM-MSCs, as indicated by increased deposition of sulfated proteoglycans (as assessed by Alcian blue staining) and significant upregulation of the chondrogenic markers *COL2A1*, and *ACAN* with reduced expression of *COL1A1* (Figure 1D). Conversely, T1-iMSCs displayed decreased extracellular matrix (ECM) calcification and expression of the osteoblast master transcription factor *RUNX2* compared with XSF-iMSCs during osteogenic induction (Figure 1E). The osteogenic culture of BM-MSCs showed high expression of the osteoblast marker *SP7* but decreased expression of *BGLAP*, a *bona fide* marker for mature osteoblasts. This observation could potentially account for the extensive alizarin red staining, but fewer dark calcified areas when comparing to iMSC osteogenic cultures. When subjected to adipogenic differentiation, T1-iMSCs synthesized fewer lipid vacuoles and showed decreased mRNA expression of the adipogenic markers *CEBPA* and *FABP4* compared with XSF-iMSCs (Figure 1F). In contrast, the early adipogenic marker *CEBPB* was significantly upregulated in T1-iMSCs. BM-MSCs showed remarkable adipogenic potential compared to iMSCs as evidenced by greater lipid production and the upregulation of the major adipogenic markers. These data suggest that although the iMSCs were derived from common progenitor cells and showed indistinguishable characteristics by conventional characterization methods, the differentiation potential was differently modulated by the medium composition during iMSCs differentiation. Notably, T1-iMSCs selectively gained a greater chondrogenic potential during expansion.

### 2.2 Transforming growth factor-β (TGF-β) signaling pathway positively correlates with iMSC susceptibility to chondrogenesis

**FIGURE 2:**
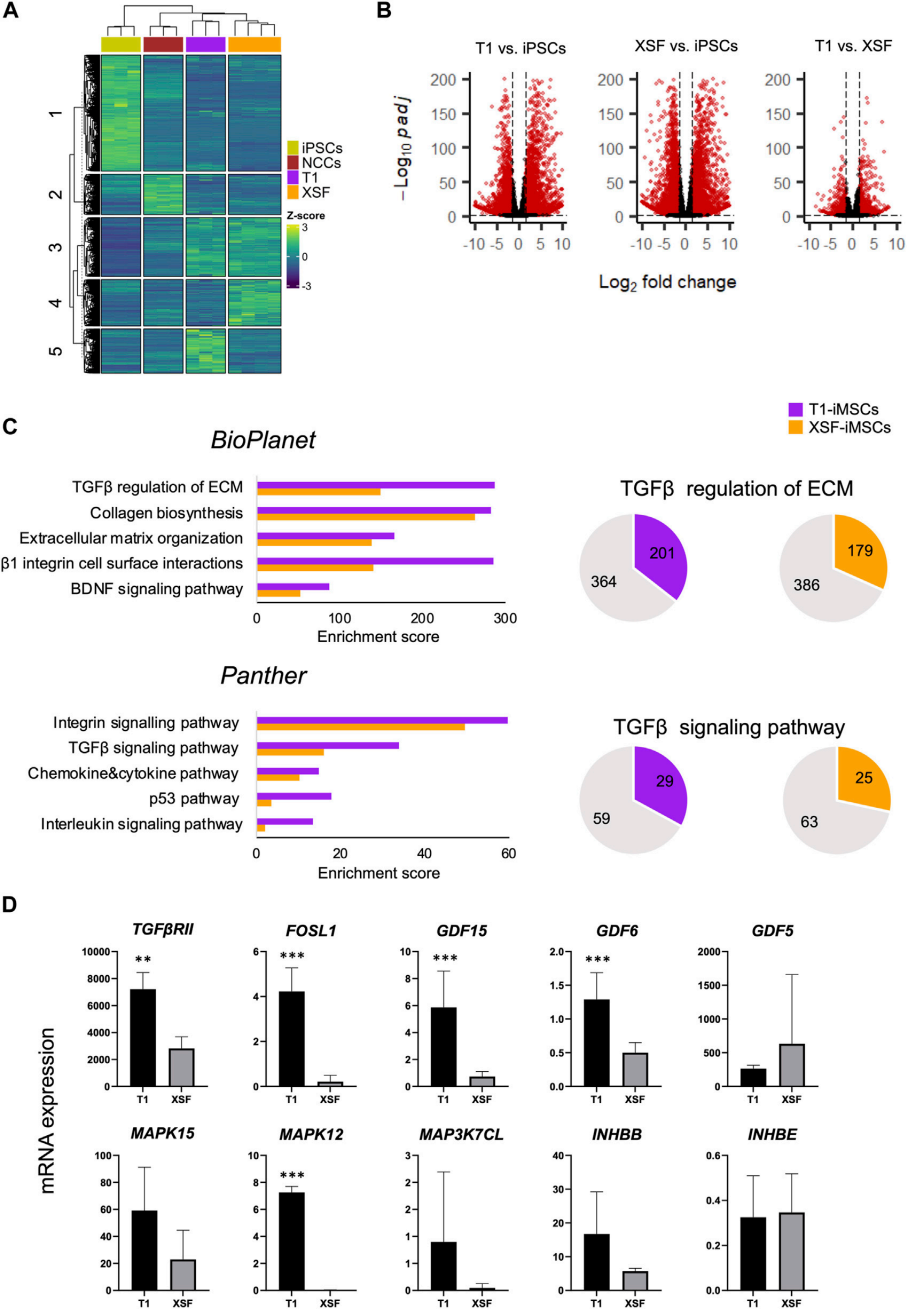
Global gene expression profile of iMSCs and parental iPSCs **(A)** Heatmap illustrating the count data of the RNAseq results and k-means clustering of the parental iPSCs (*n* = 3), iNCCs (*n* = 3), T1-iMSCs (*n* = 3) and XSF-iMSCs (*n* = 4). **(B)** Volcano plots of the differentially expressed genes as indicated. Red color dots show DEGs (*p* ≤ 0.05) that are transcriptionally up- or down-regulated (log2Fold change ≥1.5 or ≤ −1.5, respectively) **(C)** Top five enriched pathways in T1-iMSCs *versus* iPSCs (T1), and XSF-iMSCs *versus* iPSCs (XSF) (left) as well as graphic representation of the overlapped genes in selected pathways. **(D)** The mRNA expression of identified genes from the TGF-β pathway in T1-iMSCs (T1) and XSF-iMSCs (XSF) determined by RT-qPCR. Data represent the means ± SD of three independent experiments (*n* = 3). **p < 0.01, ***p < 0.005 as indicated.

To elucidate the differences between iMSCs leading to improved chondrogenesis, we performed a comparative global gene expression analysis of parental iPSCs, iNCCs, T1-iMSCs, and XSF-iMSCs using next-generation RNA sequencing (RNAseq). Figure 2A shows a heatmap of the normalized counts, highlighting the transcriptomic differences between the samples. Five clusters were identified by k-means clustering. Cluster 1 included genes that were specifically upregulated in iPSCs, while cluster 2 comprised genes upregulated only in iNCCs. Cluster 3 showed genes that were upregulated in both iMSCs but not in iNCCs or iPSCs. Importantly, cluster 4 & cluster 5 revealed a different set of upregulated genes in XSF-iMSC and T1-iMSC, respectively. To further investigate these differences, we performed a differential gene expression analysis. Figure 2B shows volcano plots of the significantly differentially expressed genes (DEGs) in T1-iMSCs vs. iPSCs (left panel), XSF-iMSCs vs. iPSCs (middle panel), and T1-iMSCs vs. XSF-iMSCs (right panel) with *p*-value ≤0.05. To further narrow down our analysis, we restricted the threshold to log2 fold-change greater than 1.5 with *p*-value ≤0.05 (red dots in Figure 2B). We then extracted the list of DEGs upregulated in T1-iMSCs vs. iPSCs (log2 fold-change ≥1.5) and performed a pathway analysis using EnrichR (Chen et al., 2013; Kuleshov et al., 2016; Xie et al., 2021)*,* a gene set search engine. The same procedure was followed for the XSF-iMSC *versus* iPSCs dataset. Figure 2C shows a plot of the top five enriched pathways by the combined score obtained from two gene set libraries, BioPlanet (Huang et al., 2019) and Panther (Thomas et al., 2022). Results from both libraries showed enrichment of TGFβ-related pathways known to play a key role in chondrogenic differentiation of MSCs. Notably, T1-iMSCs showed greater enrichment scores for these terms compared to XSF-iMSCs. Enrichment of integrin-related signaling pathways, recently proposed to regulate various chondrocyte functions such as differentiation and matrix production (Knudson and Loeser, 2002), was also found to be superior in T1-iMSCs.

Therefore, we performed a pathway enrichment analysis of DEGs upregulated in T1-iMSCs *versus* XSF-iMSCs. Consistently, the TGF-β and integrin signaling pathways were among the top 10 enriched terms by the combined score (Supplementary Table S1). Given the relevance of TGF-β in chondrogenesis, we decided to confirm the results by extracting the overlapping genes with the TGF-β pathway and evaluating mRNA expression in T1-iMSCs and XSF-iMSCs. Figure 2D shows that out of the ten identified genes, eight were upregulated in T1-iMSCs. Overall, these data suggest that the susceptibility of T1-iMSCs to chondrogenesis may be mediated, at least partially, through the TGF-β signaling pathway. The integrin signaling pathway may also contribute to these findings.

### 2.3 The small molecule TD-198946 and a stepwise differentiation strategy widely enhances the chondrogenic phenotype in T1-iMSCs

**FIGURE 3:**
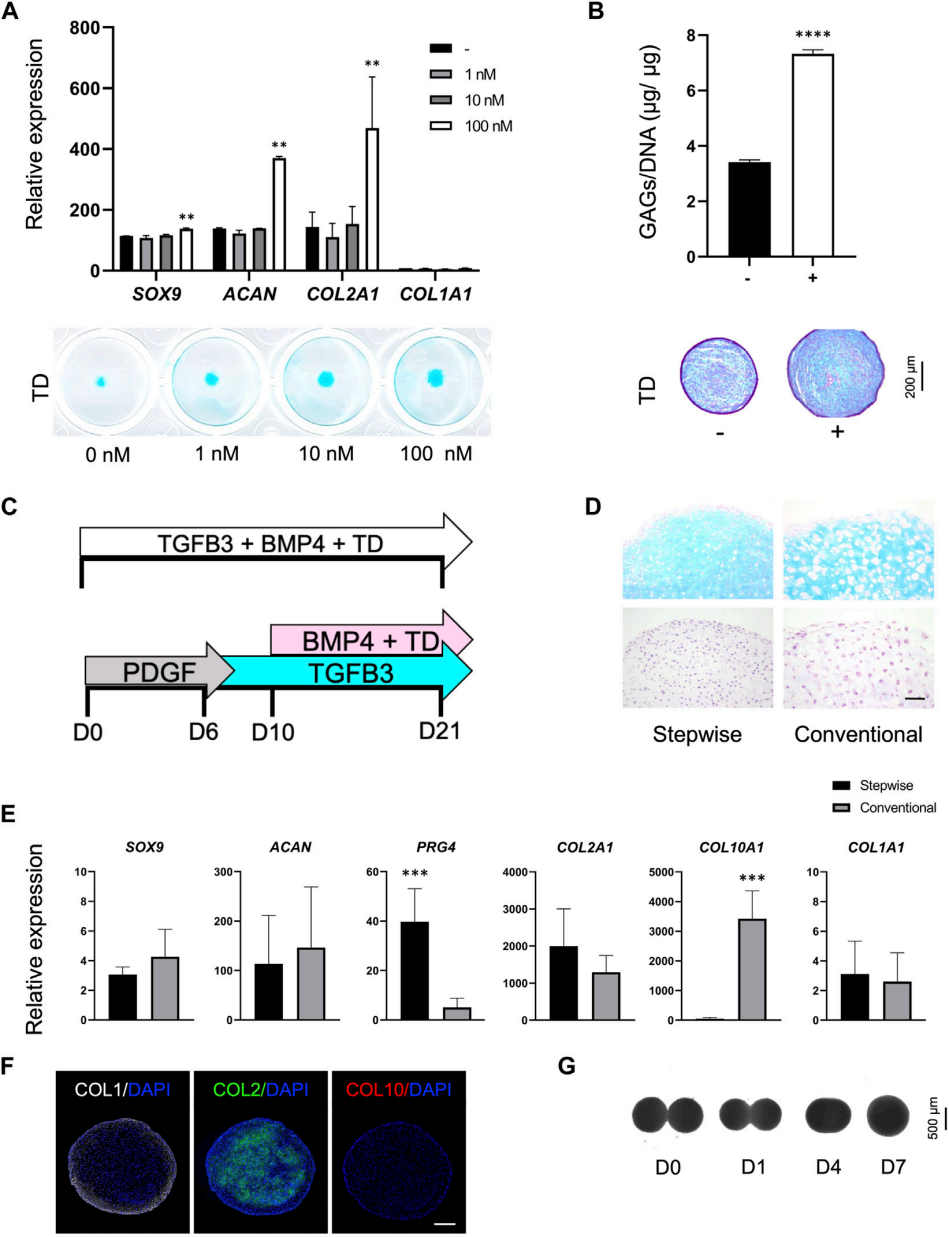
Optimization of the chondrogenic differentiation strategy **(A)** mRNA expression levels of chondrogenic markers in T1-iMSCs subjected to chondrogenic differentiation using different concentrations of TD determined by RT-qPCR and representative images of Alcian blue staining. **(B)** Quantification of GAG content (*n* = 4) and representative images of T1-iMSC chondrogenic spheroids stained with Alcian blue on day 21, differentiated in the presence or absence of TD (100 nM). **(C)** Schematic diagram of the differentiation strategies for the induction of chondrogenesis. **(D)** Histological analysis of chondrogenic spheroids on day 21 generated from T1-iMSCs using different strategies. H&E staining (upper panel) and Alcian blue staining (lower panel) is shown. Scale bar: 50 μm. **(E)** mRNA expression levels of chondrogenic markers determined by RT-qPCR in T1-iMSCs subjected to chondrogenic differentiation using different strategies. **(F)** Immunostaining of chondrogenic spheroids for COL1, COL2, and COL10 on day 21 differentiated using the stepwise strategy from T1-iMSCs. Scale bar: 100 μm. **(G)** Representative images of the fusion process of two chondrogenic spheroids. mRNA data represent the means ± SD of three independent experiments (*n* = 3). **p < 0.01, ***p < 0.005, ****p < 0.001 as indicated.

To generate an efficient chondrogenic differentiation method, we first examined whether the thienoindazole derivative TD-198946 (TD) could enhance chondrogenesis and synthesis of cartilage ECM. T1-iMSCs were treated with different concentrations of TD (1, 10, and 100 nM) in micromass cultures. Figure 3A shows that T1-iMSCs treated with TD at 100 nM showed increased expression of *SOX9*, *ACAN*, and *COL2A1* compared to all other concentrations tested, with no significant effect on *COL1A1* expression. Alcian blue staining also showed increased sulfated ECM deposition following TD treatment in a concentration-dependent manner. Given the positive effect of TD on chondrogenesis, we investigated whether the addition of TD (100 nM) during chondrogenic differentiation may alter the observed differences between T1-iMSCs and XSF-iMSCs. The chondrogenic potential of T1-iMSCs remained higher even in the presence of TD (Supplementary Figure S1).

Next, we examined whether TD added to chondrogenic medium could enhance differentiation of T1-iMSCs in 3D spheroid cultures in the presence BMP4 known for its potent chondrogenic effect (Miljkovic et al., 2008). Spheroids treated with 100 nM showed enhanced production of sulfated proteoglycans and glycosaminoglycans (GAGs) compared to untreated ones, as indicated by a quantitative dye-binding assay and Alcian blue staining (Figure 3B).

Chondrogenic differentiation of MSCs is traditionally performed by sustained stimulation with factors such as TGF-β and bone morphogenetic proteins (BMPs). Recently, stepwise differentiation strategies have emerged (Nakayama et al., 2003; Umeda et al., 2012). Therefore, we aimed to compare the chondrogenic phenotype of T1-iMSCs cultured using the conventional protocol *versus* a stepwise differentiation strategy in the presence of 100 nM TD, as depicted in Figure 3C. The small molecule TD was added during the BMP4 stimulation period, as previous reports have shown that TD acts in a BMP-dependent manner (Supplementary Figure S2).

Histological analysis revealed that the stepwise differentiated spheroids showed richer sulfated proteoglycans ECM than those differentiated using the conventional method (Figure 3D). Increased cell size, a characteristic commonly observed in hypertrophic chondrocytes, was detected in spheroids produced via the conventional method. *SOX9*, *ACAN*, *COL2A1*, and *COL1A1* were expressed at similar levels under both culture conditions. However, *PRG4*, a marker expressed exclusively in the superficial zone of articular cartilage, was upregulated in the differentiated spheroids using the stepwise strategy, whereas *COL10A1*, a marker for hypertrophic chondrocytes, was upregulated by the conventional differentiation method (Figure 3E). Furthermore, immunohistochemistry in the spheroids differentiated in a stepwise manner consisted of a COL2A1-rich inner matrix surrounded by a COL1A1-rich layer, while COL10A1 was not detected (Figure 3F). In contrast, spheroids from iMSCs or BM-MSCs differentiated with the conventional method showed limited COL2A1. The expression of COL1A1 was restricted to the outer layer and COL10A1 was detected only in spheroids derived from BM-MSCs (Supplementary Figure S3). Finally, the iMSC chondrogenic spheroids generated with the optimized stepwise strategy also had the potential to fuse completely within 7 days (Figure 3G, Supplementary Video S1).

### 2.4 Chondrogenic spheroids maintain the phenotype and do not undergo endochondral ossification *in vivo*

**FIGURE 4:**
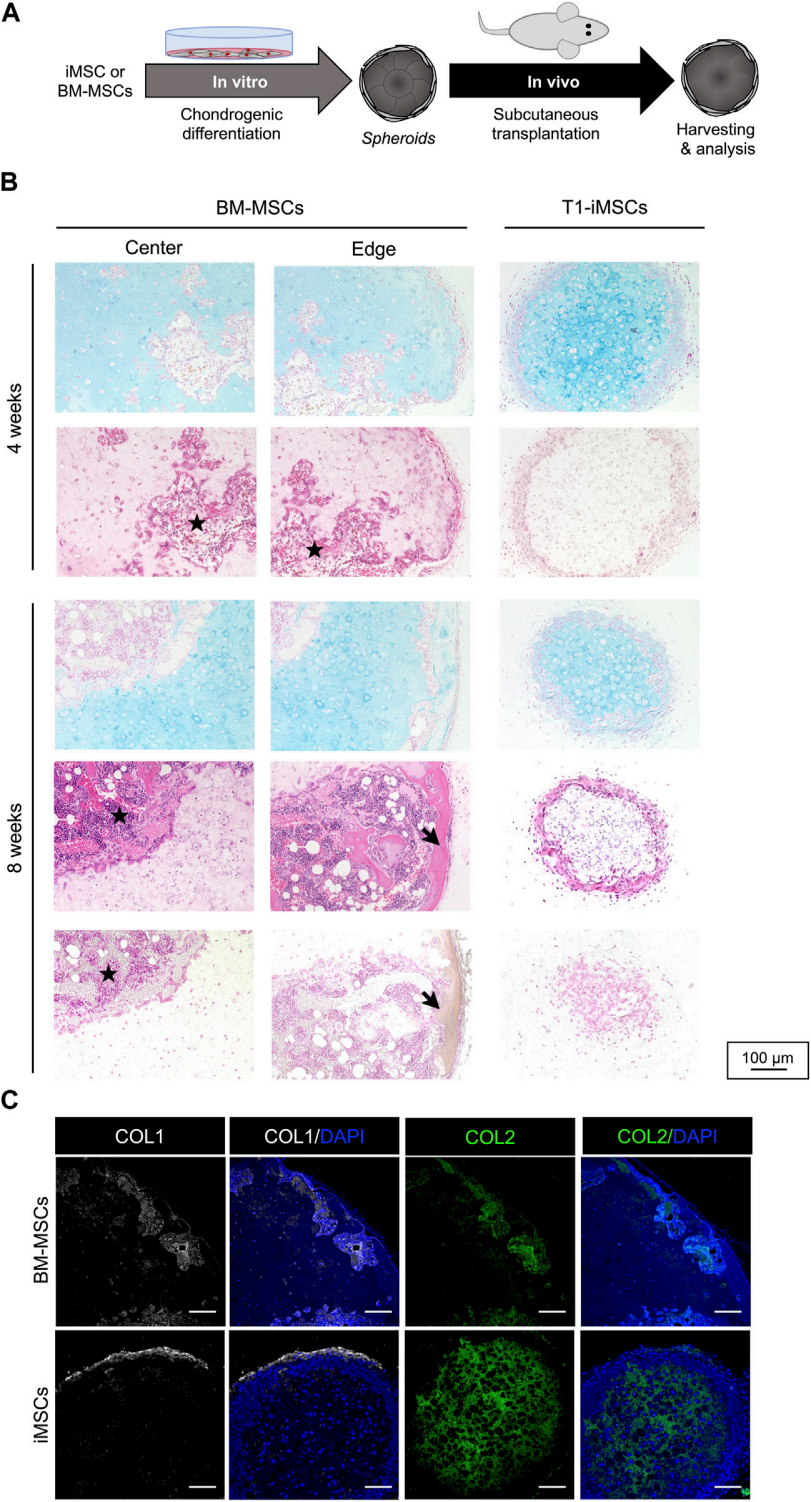
Subcutaneous transplantation of chondrogenic spheroids **(A)** Schematic diagram of the experimental procedure. **(B)** Histological analysis of the retrieved spheroids after four (upper panel) and eight (lower panel) weeks. Representative pictures of Alcian blue staining (top), H&E staining (middle), and von Kossa staining (bottom). Stars represents potential cell infiltration of the host cells and arrows indicate calcified areas. **(C)** Immunostaining of COL1 and COL2 in the retrieved BM-MSC and T1-iMSC chondrogenic spheroids at 8 weeks. Scale bar: 100 μm.

Given the potential of T1-iMSCs to form cartilage-like tissues *in vitro*, we determined their ability to maintain chondrogenic phenotype *in vivo*. Following *in vitro* differentiation, the cell spheroids were subcutaneously transplanted into immunodeficient mice (Figure 4A). Chondrogenic spheroids derived from BM-MSCs were used as positive controls to study endochondral ossification. The spheroids were harvested after four and 8 weeks for further analysis. Figure 4B shows that vascular penetration was detected within the cartilage matrix in the retrieved BM-MSCs spheroids after 4 weeks. In contrast, chondrogenic spheroids from T1-iMSCs did not show cell infiltration in the host, and Alcian blue staining revealed a homogeneous cartilage-like matrix. After 8 weeks, developing bone-like tissue was clearly identified in BM-MSCs spheroids by a well-defined edge between the remaining cartilage-like tissue and the calcified tissue that was positive for von Kossa staining. The iMSC spheroids remained as cartilage, although the size decreased, and the lacunae appeared enlarged compared to those maintained *in vitro*. Unlike BM-MSC derived spheroids retrieved at 8 weeks, the spheroids from iMSCs still showed a COL2A1-rich inner matrix surrounded by a COL1A1 layer, suggesting the maintenance of stable cartilage-like tissue *in vivo* (Figure 4C).

## 3 Discussion

The repair of articular cartilage defects using regenerative medicine requires suitable cell sources and the establishment of effective expansion and differentiation methods. In this study, we established a stepwise differentiation strategy to generate stable matrix-rich chondrogenic spheroids from iMSCs via induction of neural crest cells under defined conditions. Our approach included the generation and selection of iMSCs with a greater potential for chondrogenesis and optimization of the differentiation strategy.

The generation and cryopreservation of cells that cannot be isolated from adult tissues constitute the unique advantage of using iPSCs for cell-based therapy and *in vitro* studies of various skeletal tissues. We demonstrated that cryopreserved iNCCs could be differentiated into functional iMSCs and expanded with selectively enhanced chondrogenic potential using a commercially available xeno-free culture medium. It is known that cell culture media and expansion conditions influence the properties of tissue-derived MSCs and can prime the cells to a specific cell type by promoting or suppressing important factors regulating lineage-specific gene expression (Baer et al., 2010; Hudson et al., 2011; Gharibi and Hughes, 2012; Liu et al., 2017; Noronha et al., 2019). Under our culture conditions, enrichment of TGF-β and integrin-related signaling are hallmarks of pro-chondrogenic T1-iMSCs.

*In vitro* and *in vivo* studies have shown that TGF-β plays essential roles at all stages of chondrogenesis (Li et al., 2005; Wang et al., 2014). Initially, MSC condensation, which is required for chondrogenesis, was promoted by TGFβ-induced upregulation of N-cadherin and fibronectin expression (Tuli et al., 2003; Song et al., 2007). TGF-β has been shown to support chondrogenic differentiation of MSCs, partially through stimulatory activities on MAP kinases and modulation of Wnt signaling (Fischer et al., 2002; Tuli et al., 2003). In particular, p38 MAPK signaling was found to regulate chondrocyte-specific genes by mediating the interaction between TGF-β1 and Smad1/4 molecules in BM-MSCs (Ma et al., 2019). RNAseq analysis of the DEGs in T1-iMSCs vs. XSF-iMSCs identified the p38 MAPK pathway (P05918) and the cadherin signaling pathway (P00012) within the top ten enriched terms (Supplementary Table S1). Following chondrogenesis, TGF-β signaling is also positively correlated with chondrocyte proliferation and ECM deposition, while it inhibits terminal differentiation into hypertrophic chondrocytes (Yang et al., 2001; Li et al., 2005; Wang et al., 2014). TGF-β is the only known effective inducer of chondrogenic activity in cultured MSCs (Somoza et al., 2018). For instance, a brief pretreatment with TGF-β increased the precartilaginous condensing capacity of ectomesenchymal cells generated from iNCCs expanded under conditions similar to ours (Umeda et al., 2015). Integrins are transmembrane cell surface receptors that interact with the ECM and regulate cell functions including adhesion, migration, and differentiation. Integrin-mediated mechanisms can stimulate intracellular signal transduction in MSCs. The transition from collagen type I to type II is correlated with a switch from α1 integrin to α3 integrin during MSC chondrogenic differentiation (Shakibaei et al., 1995). Likewise, deregulation of integrin signaling is associated with osteoarthritis (Knudson and Loeser, 2002). Transcriptome-wide analyses of high-quality articular-like cartilage pellets derived from human neonatal articular cartilage cells showed strong enrichment of integrin pathways compared to chondrogenic MSC-derived pellets with limited chondrogenic potential (Somoza et al., 2018). Furthermore, growing evidence demonstrates crosstalk between the integrin and TGF-β pathways in cartilage (Munger and Sheppard, 2011).

According to our analysis, it is likely that soluble factors present in T1 medium can stimulate both TGF-β and integrin signaling. It is possible that TGF-β signaling is activated indirectly by an integrin-mediated pathway. It is known that TGF-β must be released from its latent complex to interact with its cell surface receptors, and integrins are central to this process in at least two of the three TGF-β isoforms. We also observed significant upregulation of TGFβRII in T1-iMSCs compared to that in XSF-iMSCs, suggesting that TGF-β induces rapid translocation of its own receptors to the cell surface, thus amplifying its own response as shown in previous report (Duan and Derynck, 2019). Expression of GDF15 and GDF6 but not GDF5 was higher in T1-iMSCs compared to XSF-iMSCs. The effect of GDFs, and their functions on chondrogenesis are still largely unknown compared with other members of TGF-beta super family. Functional differences among GDFs should be also further studied. For instants, GDF6 stimulation of MSCs resulted in a significant increase in a higher aggrecan and collagen type II gene expression, and higher GAG production compared with TGF-β or GDF5 stimulation (Clarke et al., 2014). Therefore, several aspects of the nature of T1 medium stimulation remain unclear and will be the subject of future investigation in our laboratory.

One limitation of this study is that the composition of T1 and XSF media is largely unknown; it was disclosed that T1 medium contains dexamethasone (Dex) at 90 nM concentration along with 0.2 μg/ml recombinant human Laminin-511 E8 fragment (iMatrix). Although Dex is a common chemical factor used for the differentiation of MSCs, lineage specification is highly dependent on the concentration used and other specific culture conditions. For example, Dex can promote adipocyte differentiation by upregulating *C/EBPα* expression but inhibits adipogenesis via *RUNX2* (Zhou et al., 2019). Dex used at concentrations below 10 nM promotes chondrogenesis in human synovial MSCs but attenuates chondrogenesis at concentrations higher than 100 nM (Chijimatsu et al., 2018). Moreover, low-dose Dex treatment (10^–8^ M) preserves the stemness of expanded human MSCs (Xiao et al., 2010). In contrast, laminins promote adhesion and expansion of various cells, including embryonic stem cells, iPSCs, and MSCs (Miyazaki et al., 2012). Further studies have shown that laminins participate in chondrogenic differentiation by upregulating *COL2A1* expression in human chondrogenic progenitor cells and GAG content in human MSCs (Schminke et al., 2016; Sun et al., 2017). Although the exact mechanisms by which laminins influence stem cells are complex, they involve interactions with integrins, which can bind with broad specificity and high affinities. In particular, laminin-511 exhibited the highest affinity for α6 integrins (ITGA6). Notably, *ITGA6* was upregulated among the DEGs in T1-iMSCs *versus* XSF-iMSCs.

Protocols to induce chondrogenesis in MSCs *in vitro* coincide in two aspects: high cell density (pellets or micromasses) and stimulation with TGF-β1 or TGF-β3. We hypothesized that T1-iMSCs with greater susceptibility to integrin and TGF-β signaling account for their increased chondrogenic potential when subjected to further differentiation. Moreover, our results suggest that the expansion and generation of iMSCs in the presence of Dex and iMatrix may be a powerful approach for generating an MSC population with enhanced chondrogenic potential.

TD has been shown to be a chondrogenic factor as potent as insulin, BMP2, and TGF-β1 (Saito et al., 2013). Administration of TD also induces cartilage regeneration in murine models of osteoarthritis (Yano et al., 2013a) and intervertebral disc degeneration (Chijimatsu et al., 2019) *in vivo*. Here, we show for the first time the chondrogenic potential of TD on iPSC-derived MSCs. Although the exact mechanism by which TD supports chondrogenesis is unclear, TD has been reported to upregulate *Runx1*, *Sox9*, and *Col2a1* (Yano et al., 2013a; Hamamoto et al., 2020). T1-iMSCs treated with 100 nM TD showed upregulation of *SOX9*, *ACAN*, and *COL2A1* as well as increased GAG production without a noticeable effect on hypertrophy. Consistent with previous reports, TD and BMP stimulation had a positive synergistic effect on chondrogenesis. These findings could potentially contribute to the use of TD in regenerative medicine using iPSC-derived chondrogenic progenitors. Finally, we demonstrated that more relevant protocols are required to efficiently induce chondrogenesis in 3D aggregates. In particular, the traditional method of continuously stimulating cells with chondrogenic factors such as TGF-βs and BMPs often produces hypertrophic phenotypes and a less rich cartilage matrix. In contrast, the stepwise differentiation approach with a sequential transition from platelet-derived growth factor to TGF-β3 and BMP4 (Umeda et al., 2012) in combination with TD molecules, formed spheroids with stable cartilage-like tissue that was resistant to vascular invasion and calcification *in vivo*. Given the potential of the generated spheroids to fuse within a few days, our method is compatible with modern tools for tissue engineering such as bioprinting using the Kenzan method (Nakayama, 2013; Nakamura et al., 2021).

Overall, this study presents a novel method for producing and expanding clinically relevant pro-chondrogenic iMSCs as well as an improved chondrogenic differentiation strategy for the stable generation of cartilage-like tissue. The insights provided here will help identify and select stem cell progenitors with superior chondrogenic potential for regenerative medicine and cartilage tissue engineering.

## 4 Methods

### 4.1 Generation, expansion, and cryopreservation of iNCCs from iPSCs under xeno-free conditions

Human iPSCs 1231A3 reprogrammed with episomal vectors (kindly provided by Yamanaka Laboratory) were maintained as described previously (Nakagawa et al., 2014). For NCC induction, 3 × 10^4^ cells/well were seeded in 6-well plates coated with iMatrix-511 (Nippi, Tokyo, Japan) in StemFit AK03N medium (Ajinomoto, Tokyo, Japan). After 4 days, the medium was replaced with NCC induction medium containing 10 μM SB431542 (FUJIFILM Wako Pure Chemical Corporation, Japan) and 1 μM CHIR99021 (Axon Medchem, Reston, VA, United States), as described previously (Kamiya et al., 2022). On day 10, CD271high positive cells were sorted and replated on fibronectin-coated plates at a density of 1 × 10^4^ cells/cm^2^ in NCC expansion medium: Basic03 medium supplemented with 10 μM SB431542, 20 ng/ml EGF (FUJIFILM Wako Pure Chemical Corp.), and 20 ng/ml FGF2 (FUJIFILM Wako Pure Chemical Corp.). The medium was changed every 2–3 days. For cell passage, the cells were dissociated with Accutase (Innovative Cell Technologies, San Diego, CA, United States). iNCC stocks were prepared using STEM-CELLBANKER GMP grade (Takara Bio Inc., Kusatsu, Japan).

### 4.2 Differentiation of cryopreserved iNCCs into iMSCs under xeno-free conditions

iNCCs from frozen stocks at passage number (PN2) were cultured and expanded to PN5 in fibronectin-coated plates. Then, the medium was replaced with either PRIME-XV MSC Expansion XSFM (FUJIFILM Irvine Scientific, Tokyo, Japan) to generate XSF-iMSCs or T1 medium composed of StemFit For MSC (Ajinomoto, Tokyo, Japan) supplemented with 90 nM Dex and 0.2 µg/mL iMatrix-511 to generate T1-iMSCs. Cells were transferred to fibronectin-coated plates at a density of 1 × 10^4^ cells/cm^2^ up to seven times before further differentiation. iMSCs stocks in PN2 were prepared using the STEM-CELLBANKER GMP grade.

### 4.3 Real-time quantitative PCR analysis

Total RNA was purified using the RNeasy Micro Kit (Qiagen, Hilden, Germany) and reverse-transcribed to cDNA. Real-time quantitative PCR (RT-qPCR) was performed using THUNDERBIRD™ Next SYBR^®^ qPCR mix (QPX-201; Toyobo Co., Ltd.), QuantStudio™ 3 Real-Time PCR System, and QuantStudio™ 7 Flex Real-Time PCR System (Applied Biosystems, Waltham, MA, United States). Primer sequences are summarized in Supplementary Table S2. Data from at least three biological replicates were analyzed to calculate the relative fold-change (2^−ΔΔCT^). All data were plotted as fold change relative to iPSCs using GraphPad Prism 9 software.

### 4.4 Flow cytometry analysis

T1-iMSCs and XSF-iMSCs (PN3–PN4) were stained on ice for 30 min with the following antibodies (1:50 dilution in FACS buffer): CD105-APC (eBioscience, 17-1057-42), CD90-PE (eBioscience, 555596), CD73-PE (eBioscience, 550257), CD44-PE (eBioscience, 550989), CD105APC (eBioscience, 17-1057-42), PE-isotype (BD Bioscience, 551438), and APC isotype (BD Bioscience, 565381). After washing, BD FACSAria™ III Cell Sorter was used to detect the fluorescence. The results were plotted using FlowJo_v10.8.1.

### 4.5 Three lineage differentiation of iMSCs

The same procedure was followed for the differentiation of T1-iMSCs and XSF-iMSCs (collectively called iMSCs), and all experiments were carried out in parallel with cells at the same passage number (PN4–PN7). As a reference, human BM-MSCs (PT-2501, (Batch 20TL262529; Lonza, Durham, NC, United States) at PN5 were also differentiated under the same conditions. Chondrogenic differentiation was performed using micromass culture onto fibronectin-coated 24-well plates. Briefly, 1.5 × 10^5^ iMSCs were resuspended in 5 µL of chondrogenic medium consisting of DMEM/F12 (Thermo Fisher Scientific, Waltham, MA, United States), 1% (v/v) ITS + premix (Corning, Corning, NY, United States), 0.17 mM AA2P (Sigma-Aldrich, St. Louis, MO, United States), 0.35 mM Proline (Sigma-Aldrich), 0.1 mM Dex (Sigma-Aldrich), 0.15% (v/v) glucose (Sigma-Aldrich), 1 mM sodium-pyruvate (Thermo Fisher Scientific), and 2 mM GlutaMAX (Thermo Fisher Scientific) supplemented with 10 ng/ml TGF-β3 (R&D Systems, Minneapolis, MN, United States). BMP7, commonly used for 2D chondrogenic cultures (Caron et al., 2013), was also added to the differentiation medium at 50 ng/ml (R&D Systems). After 1 h incubation, 1 ml of chondrogenic medium was added to each well and the cells were cultured for 7 days. Chondrogenesis was assessed by Alcian blue staining. Briefly, induced cells were fixed for 30 min with 4% paraformaldehyde (PFA) (FUJIFILM Wako Pure Chemicals Corp.) and rinsed with phosphate buffered saline. The cells were then incubated with 1% Alcian Blue solution (Muto Pure Chemicals Co., Ltd., Tokyo, Japan) for 1 h at room temperature and washed five times with phosphate buffered saline before imaging. For osteogenic differentiation, 5 × 10^4^ iMSCs were seeded onto gelatin-coated wells, maintained until they reached full confluence, and then cultured in osteogenic induction medium containing MEM-Alpha GlutaMAX (Gibco, 32571-036), 10% fetal bovine serum (Thermo Fisher Scientific), 0.5% penicillin/streptomycin, β-glycerophosphate disodium salt hydrate (Sigma-Aldrich, G9422), and 100 nM Dex. The medium was changed every 2–3 days. After 3 weeks, the PFA-fixed cells were stained with Alizarin Red S solution (Muto Pure Chemicals Co., Ltd., 17971). For adipogenic differentiation, 5 × 10^4^ iMSCs were seeded on fibronectin-coated 12-well plates, and adipogenic induction was initiated when they reached full confluence by replacing the MSC medium with adipogenic induction medium containing DMEM (08459-64, High Glucose; Nacalai Tesque, Japan), 10% fetal bovine serum (Nichirei, 171012), 0.5% penicillin/streptomycin (Gibco, 15140122), 10 μg/ml insulin (Wako Pure Chemicals Corp., 097-06474), 1 µM Dex (Wako Pure Chemicals Corp., 047-18863), 200 µM indomethacin (Wako Pure Chemicals Corp., 093-02473), and 500 µM IBMX (Wako Pure Chemicals Corp., 095-03413). PFA-fixed cells were washed with water, incubated with 60% isopropanol for 5 min, and stained with Oil Red O (Nacalai Tesque, 25633-92) dissolved in 60% isopropanol. Non-specific staining was removed by washing several times with water.

### 4.6 RNAseq data analysis

Total RNA was purified using an RNeasy Micro Kit (Qiagen) and treated with a DNase I kit (Qiagen) to remove genomic DNA. We reverse-transcribed 10 ng of total RNA to obtain single-stranded cDNA using the SuperScript VILO cDNA Synthesis Kit (Thermo Fisher Scientific). We synthesized cDNA libraries for the Ion Ampliseq transcriptome analysis using the Ion AmpliSeq Transcriptome Human Gene Expression Core Panel (Thermo Fisher Scientific) and Ion Ampliseq Library Kit Plus (Thermo Fisher Scientific), according to the manufacturer’s protocol. Briefly, cDNA was amplified for 12 cycles with an Ion AmpliSeqTM Transcriptome Human Gene Expression Core Panel using a thermal cycler. Primer sequences were partially digested with the FuPa reagent by sequentially performing 10 min at 50°C, 10 min at 55°C, and 20 min at 60°C. Barcode ligation was performed using an Ion Xpress Barcode for 30 min at 22°C. Barcode-labeled cDNA libraries were purified using DNA Clean & Concentrator™-5 (Zymo Research, CA, United States) and analyzed using the Ion S5 XL System (Thermo Fisher Scientific) and Ion 540 Chip Kit (Thermo Fisher Scientific). Count data analyses were performed using R studio with the “DESeq2” package normalization method for the detection of significantly (*p* ≤ 0.05) differentially expressed genes (Love et al., 2014).

### 4.7 Optimization of chondrogenic differentiation strategy

The micromass culture described in the previous section was used to evaluate the small-molecule TD-198946 (TD) (MedChemExpress, HY-15642/CS-6860) in T1-iMSCs. Different concentrations were tested, and the results are shown in Figure 3A. Subsequently, 3D culture was used to produce chondrogenic spheroids. Briefly, 2 × 10^4^ T1-iMSCs/well were plated onto ultra-low attachment 96 U-well plates (Sumitomo Bakelite Co., Ltd., Tokyo, Japan) in hMSC Chondrogenic Basal Medium (PT-3925) and hMSC Chondrogenic SingleQuots™ Kit Supplement (PT-4121). Chondrogenic inducers were added at specific time points, as indicated in Figure 3C, including 40 ng/ml platelet-derived growth factor-BB (PDGF-BB; R&D Systems), 10 ng/ml TGF-β3 (Peprotech Inc., Rocky Hill, NJ, United States), 50 ng/ml BMP4, and 100 nM TD. Cells were cultured for 21 days and the medium was changed every 3–4 days. At day 21, spheroids were isolated for mRNA expression, quantification of sulfated proteoglycans and GAGs, immunostaining, and histological analysis.

### 4.8 Production of sulfated proteoglycans & GAGs

Chondrogenic spheroids were collected on day 21 and digested for 6 h in 50 µg/ml papain solution (Sigma-Aldrich) at 65°C. The obtained extracts were used to quantify the GAG content using a Blyscan™—sulfated glycosaminoglycan (sGAG) assay kit, following the manufacturer’s instructions. DNA content was determined using Quant-iT™ PicoGreen ^®^ dsDNA Assay Kits and dsDNA Reagents (Invitrogen, Waltham, MA, United States).

### 4.9 Fusion experiment

A set of two chondrogenic spheroids (day 21) per well was placed in 96 U-well plates and cultured in chondrogenic medium for an additional week. The medium was replaced on day 4. Images were obtained on days 0, 1, 4, and 7. To enable accurate monitoring of the fusion process while maintaining stable environmental conditions, we used an automated system for capturing images every 30 min during the first 4 days (BioStation CT, Nikon). The images were combined to produce a video (Supplementary Video S1).

### 4.10 Immunocytochemistry

Prior to immunostaining, paraffin sections or cryosections of PFA-fixed spheroids were prepared. Staining was performed as previously reported (Zujur et al., 2017) and DAPI (1:1000; Thermo Fisher Scientific) was used to counterstain nuclei. The primary antibodies used in this study are listed in Supplementary Table S3. Observations and assessments of the samples were performed using a BZ-X700 Fluorescence Microscope (Keyence, Osaka, Japan) or Olympus FV3000 confocal laser scanning microscope.

### 4.11 Subcutaneous transplantation of chondrogenic spheroids

Chondrogenic spheroids (day 21) of approximately 500 μm generated from iMSCs or human BM-MSCs (PT-2501, (Batch 20TL262529; Lonza, Durham, NC, United States) were subcutaneously transplanted into a small incision on the back of eight-week-old female CB17/IcrJcl-Prkdc^scid^ mice purchased from CLEA Japan, Inc. (Tokyo, Japan) (*n* = 6 per group). For this procedure, the mice were anesthetized with 3% forane inhalant liquid (AbbVie, North Chicago, IL, United States). Ethical approval was obtained from the Animal Care Committee of Kyoto University (16-73-13). The mice were sacrificed after four or 8 weeks, and the spheroids were retrieved for further analysis. The calcium stain kit of modified von Kossa (ScyTek Laboratories, UT, United States) was used following the manufacturer’s instructions for the visualization of calcium deposits in paraffin sections.

### 4.12 Statistical analysis

The means of groups were compared by analysis of variance using GraphPad Prism 9 software. Significance of differences was determined by Dunnett’s test in the case of multiple group comparisons to a single control.

## Data Availability

RNA-seq data presented in the study was deposited in the Gene Expression Omnibus (GEO) database. Accession numbers GSE230303 (overall normalized data and the raw data corresponding the iMSCs) and GSE206048 (raw data of the iPSC and iNCC samples).

## Abbreviations

BMPs, bone morphogenetic proteins (BMPs); BM-MSCs, bone marrow-derived MSCs (BM-MSCs); Dex, dexamethasone (Dex); ECM, extracellular matrix (ECM); DEGs, differentially expressed genes (DEGs); iMSCs, induced pluripotent stem cell-derived mesenchymal stem/stromal cells (iMSCs); iPSCs, Induced pluripotent stem cells (iPSCs); MSCs, mesenchymal stromal/stem cells (MSCs); iNCC, iPSC-derived neural crest-like cells (iNCC); TGF-β, Transforming growth factor-β (TGF-β); RNAseq, RNA sequencing (RNAseq); TD, TD-198946 (TD); GAGs, glycosaminoglycans (GAGs).
