# Supplementary material for: Enhanced chondrogenic differentiation of iPS cell-derived mesenchymal stem/stromal cells via neural crest cell induction for hyaline cartilage repair
Source: Front Cell Dev Biol. 2023 May 10;11:1140717. doi: 10.3389/fcell.2023.1140717 (PMC10206169; doi:10.3389/fcell.2023.1140717)
Supplement: Supplementary file 1 [file DataSheet1.pdf]

Supplementary Material

Supplementary Figure 1

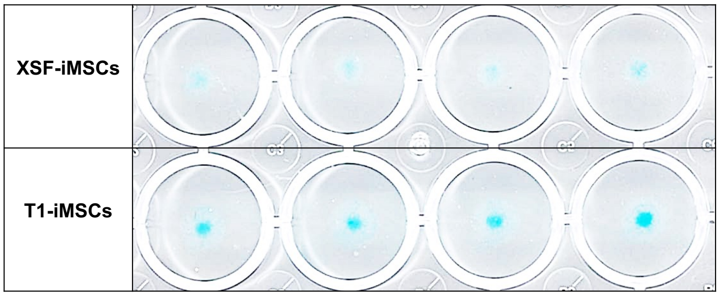

Supplementary Figure 1. Alcian blue staining of T1-iMSCs and XSF-iMSCs differentiated to chondrocytes in the presence of TD (100 nM) in micromass culture for 7 days.

Supplementary Figure 2

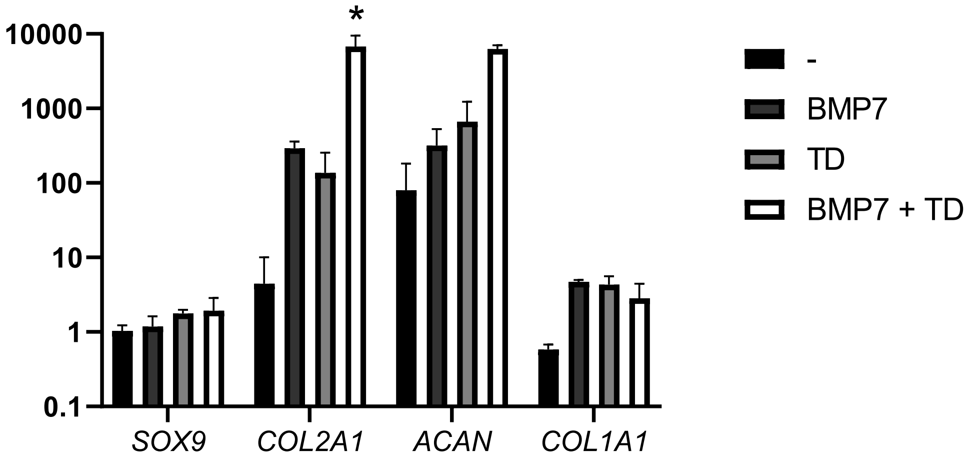

Supplementary Figure 2. The mRNA expression of chondrogenic markers determined by RT-qPCR in T1-iMSCs differentiated in micromass culture with basal chondrogenic medium (-) containing BMP7 (100 nM), TD (100 nM) or BMP7 (100 nM) + TD (100 nM). Data are the means ± SD (n=6) presented as folds relative to iPSCs. *P < 0.05 in BMP7 + TD vs. all others.

Supplementary Figure 3

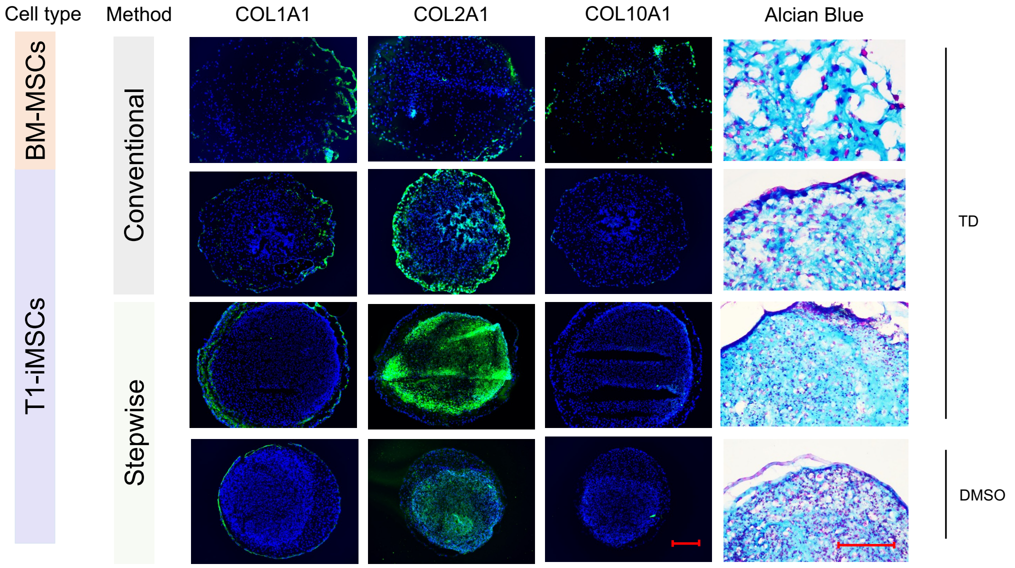

Supplementary Figure 3. Immunostaining and alcian blue staining of BM-MSC and T1-iMSC chondrogenic spheroids on day 21 generated using different strategies in the presence or absence of TD (100 nM). Scale bars: 100 μm.

Supplementary Table 1

Supplementary Table 1. Top 10 enriched terms by combined score of the DEGs in T1-iMSCs vs. XSF-iMSCs obtained from two libraries: BioPlanet and Panther.

Term	Overlap	P-value	Adjusted P-value	Odds Ratio	Combined Score
**Source: BioPlanet**					
TGF-beta regulation of extracellular matrix	76/565	1.94E-26	1.83E-23	5.1	303.5
Adipogenesis	19/133	5.89E-08	2.79E-05	5.1	84.9
BDNF signaling pathway	27/261	1.17E-07	3.68E-05	3.6	56.7
Interleukin-4 regulation of apoptosis	26/267	6.49E-07	1.54E-04	3.3	47.2
FSH regulation of apoptosis	25/263	1.66E-06	3.13E-04	3.2	42.9
Striated muscle contraction	9/38	2.61E-06	4.12E-04	9.4	120.6
Dilated cardiomyopathy	14/100	4.13E-06	5.58E-04	4.9	61.3
Arrhythmogenic right ventricular cardiomyopathy (ARVC)	12/75	4.88E-06	5.77E-04	5.8	70.7
Beta-1 integrin cell surface interactions	11/66	8.06E-06	8.47E-04	6.1	71.1
TAp63 pathway	10/55	9.32E-06	8.82E-04	6.7	77.9
**Source: Panther**					
5-Hydroxytryptamine degredation P04372	2/5	1.72E-04	0.01	19.968	92.32
Notch signaling pathway P00045	6/38	3.17E-04	0.01	5.6425	37.55
p53 pathway P00059	9/71	4.62E-04	0.01	4.3821	33.66
Plasminogen activating cascade P00050	3/15	1.29E-03	0.02	7.4961	33.46
Integrin signalling pathway P00034	15/156	3.59E-03	0.05	3.2286	27.99
CCKR signaling map ST P06959	15/165	6.36E-03	0.07	3.0335	24.44
Nic acetylcholine signaling pathway P00044	7/68	7.69E-03	0.08	3.4535	17.47
p38 MAPK pathway P05918	4/32	9.82E-03	0.08	4.2866	16.96
Cadherin signaling pathway P00012	12/150	1.15E-03	0.09	2.627	14.79
TGF-beta signaling pathway P00052	8/88	1.91E-03	0.13	3.0113	14.66

Supplementary Table 2

Supplementary Table 2. Primer sequences used for RT-qPCR

GENE NAME	FORWARD	REVERSE
ACTB	AGGTCTTTGCGGATGTCCACGT	CACCATTGGCAATGAGCGGTTC
SOX10	GAGCTGGACCGCACACCTTGGG	AACGCCCACCTCCTCGGACCTC
NGFR	CCGTTGGATTACACGGTCCA	GACAGGGATGAGGTTGTCGG
CD44	GCAATGCTTCTCAGACCACA	GAGGGGAGAGGGTAGACAGG
CD73	CAGTACCAGGGCACTATCTGG	AGTGGCCCCTTTGCTTTAAT
CD90	TGGATTAAGGATGAGGCCCG	CCTAAGTCACTCGCCATCCC
CD105	CCACTAGCCAGGTCTCGAAG	GATGCAGGAAGACACTGCTG
SOX9	ATTTCCTCCTGCCTTTGCTT	CTGGTGTTCTGAGAGGCACA
COL2A1	GGTGGCTTCCATTTCAGCTA	TACCGGTATGTTTCGTGCAG
COL1A1	GTGCTAAAGGTGCCAATGGT	CTCCTCGCTTTCCTTCCTCT
COL10A1	ACGCTGAACGATACCAAACG	GCACACCTGGTTTCCCTACA
ACAN	ACAGCTGGGGACATTAGTGG	GTGGAATGCAGAGGTGGTTT
PRG4	GAACGTGCTATAGGACCTTC	CAGACTTTGGATAAGGTCTGCC
RUNX2	CAGACCAGCAGCACTCCATA	CAGCGTCAACACCATCATTC
BGLAP	GGCAGCGAGGTAGTGAAGAG	AGCAGAGCGACACCCTAGAC
SP7	AAGCTGATCTGGTGGTGCAT	GACTCCACAAAGGGCATGAT
CEBPB	TTTGTCCAAACCAACCGCAC	CCCCCAAAAGGCTTTGTAACC
CEBPA	TATAGGCTGGGCTTCCCCTT	AGCTTTCTGGTGTGACTCGG
PPARG	CCAGAAGCCTGCATTTCTGC	CACGGAGCTGATCCCAAAGT
FABP4	TGGGCCAGGAATTTGACGAA	CACATGTACCAGGACACCCC
FOSL1	CATCAACACCATGAGTGGCA	TCAGTCTCCTGTTCACAAGGC
INHBE	GCTAGCCAAGCAGCAAATCC	CATGGTACAGGTGGTGGGAC
TGFBR2	CTCCTGTGCAGCTTCCCTC	TCCACAGGACGATGTGCAG
MAPK12	CCTTTCCAGTCCGAGCTGTT	GCTTCAGGTCCCTCAGCC
INHBB	GAAGAGGGTGGACCTCAAGC	CCGATGAGGCGGAAGTCAAT
GDF15	GCAAGAACTCAGGACGGTGA	TGGAGTCTTCGGAGTGCAAC
MAP3K7CL	CTCCCCTCTGGATTGTCAGT	CCTGGCTGTGCTGATCATGT
GDF6	CGATCTCTCGCACACTCCTC	CACACGTCGAAGACTTCCCA
GDF5	CGGTCGGCTTTCTCCTTTCA	ACTTTCAAAAGCAGCGGCAG
MAPK15	AGATACCTACTCAGGCGGCA	GATGGTCCCCAAACTCCTGG

Supplementary Table 3

Supplementary Table 3.

Antibody name	Supplier	Code	Host	Dilution
Anti-Collagen I	Novus Biologicals	NB600-408	Rabbit	1:100
Anti-Collagen II	Invitrogen	MA5-12789	Mouse	1:100
Anti-Collagen X	Invitrogen	14977-1	Mouse	1:100
anti-Rabbit IgG (H+L) Alexa Fluor 488	Thermo Fisher Scientific	A11008	Goat	1:500
anti-Mouse IgG (H+L) Alexa Fluor 488	Thermo Fisher Scientific	A11001	Goat	1:500

Supplementary Movie

Supplementary Movie 1. Four-day recording of the fusion process on chondrogenic spheroids generated with the optimized strategy
